# Hybrid model of intensive lifestyle intervention is potentially effective in patients with diabetes & obesity for post-COVID era

**DOI:** 10.3389/fendo.2022.1050527

**Published:** 2023-01-17

**Authors:** Shilton Dhaver, Marwa Al-Badri, Tareq Salah, Cara Kilroy, Jacqueline Shahar, Colleen Johnson, Jennie Votta, Christine Mitchell, Joan Beaton, Abdelrahman Khater, Karim Kibaa, Ryan McCarragher, Chandra Davis, Osama Hamdy

**Affiliations:** ^1^ Joslin Diabetes Center, Boston, MA, United States; ^2^ Harvard Medical School, Boston, MA, United States

**Keywords:** obesity, diabetes, digital health, telehealth, healthcare, telemedicine, COVID - 19, hybrid

## Abstract

The Weight Achievement and Intensive Treatment (Why WAIT) program is a 12-week multidisciplinary intensive lifestyle intervention (ILI) for patients with diabetes and obesity in real-world clinical practice that has led to long-term weight loss maintenance for up to 10 years. During COVID-19, we reported that a virtual model (VM) of the program was equally effective in reducing body weight and improving glycemic control. Here, we test a newly-introduced hybrid model (HM), to accommodate ongoing restrictions of the pandemic. We evaluated 56 participants: 18 from HM, 16 from VM and 22 from the in-person model (iPM). At 12 weeks, mean change in body weight from baseline for HM was -8.2 ± 5.0 kg; p<0.001. Mean change in A1C for HM was -0.6 ± 0.6%; p=0.002. There were no significant differences in body weight reduction (p=0.7) or A1C reduction (p=0.6) between groups. Blood pressure, lipid profile, and all other parameters showed improvements without significant differences between groups. Overall, HM is as effective as VM and iPM in reducing body weight and A1C after 12 weeks. Given its scalability, HM could be offered to more patients with diabetes and obesity who may benefit from its increased flexibility and enhanced accountability without compromising the multidisciplinary approach for a post-COVID era.

## Introduction

Diabetes mellitus has emerged as one of the most serious chronic conditions that carries a major impact on the lives and wellbeing of individuals, causing life-threatening, disabling and costly complications, while reducing life expectancy (1). It is estimated to impact 537 million people worldwide, with an estimated global prevalence of 10.5% of the world’s adult population (2). In the United States, the Center of Disease Control and Prevention (CDC) estimates that 37.3 million people have diabetes (11.3% of the US population) (3). Furthermore, around 96 million people aged 18 years or older (38% of the adult US population) are estimated to have prediabetes (3). It is projected that by 2045, the absolute number of people with diabetes will increase by 46%, with the greatest absolute growth in terms of number of persons with diabetes between 2021 and 2045 occurring in middle-income countries (1). The global health expenditure due to diabetes has considerably increased, growing from USD 232 billion in 2007 to USD 966 billion in 2021 for adults aged 20–79 years with estimated total diabetes-related health expenses reaching USD 1.03 trillion by 2030 and USD 1.05 trillion by 2045 (2). In effort to address this serious public health crisis, lifestyle modification through dietary intervention and increased physical activity is recommended. Lifestyle intervention programs have demonstrated to be effective in decreasing the incidence of T2D among individuals with prediabetes, as well as resulting in a significant improvement in cardiovascular risk factors among overweight and obese patients with T2D (4–7). We previously showed that multidisciplinary intensive lifestyle intervention (ILI) in real-world clinical practice can lead to maintenance of significant weight loss for up to 10 years and had positive impacts on major cardiovascular risk factors (8). Maintenance of weight loss was predicted by the participant’s ability to achieve ≥7% weight loss at 1 year following 12 weeks of ILI (9). Traditionally, lifestyle intervention programs have been conducted in-person but with the advent of the COVID-19 pandemic, the adoption of telehealth has dramatically accelerated; transforming both patient and provider experience. We reported that a fully virtual model (VM) of the ILI program for the same duration was equally effective in reducing body weight and improving glycemic control (10). However, in order to accommodate the prolonged restrictions of the ongoing pandemic and reintroduce infrequent face-to-face contact between patients and providers, we developed a hybrid model that combines the best aspects of both in-person and virtual care models. In this study, we tested the clinical outcomes of the hybrid model (HM) in comparison to the classic in-person model (iPM) and the VM conducted by the same multidisciplinary team. The primary endpoints of this study are the change in body weight and A1C after 12 weeks of intervention. The secondary endpoints include changes in percentage of glucose time in range (TIR) using continuous glucose monitoring (CGM), changes in blood pressure (BP), lipid profile, and number of diabetes medications after 12 weeks of intervention.

## Methods

### The Why WAIT program

The Weight Achievement and Intensive Treatment (Why WAIT) Program is a 12-week multidisciplinary intensive lifestyle intervention (ILI) program for diabetes and weight management in real-world clinical practice. The program has been implemented at Joslin Diabetes Center in Boston, MA since 2005. Full descriptions of the program have been published elsewhere (11). In brief, participants with either type 1 diabetes (T1D) or T2D and with body mass index (BMI) between 30 and 45 kg/m^2^ are included in the program after full clinical evaluation for the suitability for ILI. Participants are enrolled in groups. Each group is roughly 10-15 participants at the time. The ILI model of the program is delivered by a multidisciplinary team consisting of a diabetologist, a psychologist or behavioral therapist, a registered clinical exercise physiologist (RCEP), and a registered dietician (RD). The 12-week Why WAIT program includes the following intervention methods:

### Group education

RCEPs and RDs lead educational group sessions on the topic of diabetes and weight management and provide handouts as future reference. These sessions are conducted weekly throughout the program.

### Dietary intervention

The program RD develops personalized meal plans based on a dietary evaluation of each participant. Meal plans are based on individualized caloric reduction to 1200-1800 calories according to the Joslin Nutrition Guidelines. Meal plans include fiber intake of 14 g/1000 calories, protein intake of 1–1.5 g/kg of adjusted body weight, <35% of daily calories from fat with saturated fat <10%, and 40–45% of daily calories from carbohydrates (12). In the first six weeks of the program, participants use diabetes-specific formula (DSF) to replace breakfast and possibly a lunch or snack. After the first six weeks, breakfast and lunch options from natural food are offered instead of DSF, however participants are given the choice to continue to consume DSF if they desire. Healthy snacks of 100cal and 200cal are offered between meals. Participants are provided with dinner menus with cooking instructions.

### Medication adjustments

Diabetologists review participants’ medication at the beginning of the program. Medications that promote weight loss are encouraged if covered by participants’ insurance plans, weight-neutral medications are usually continued, but medications that are known to increase body weight are reduced (13). Participants’ glucose logs are reviewed weekly by a nurse practitioner and adjustments of doses are made if necessary.

### Exercise intervention

Stretching, aerobic, strength and core exercises are employed in this intervention. These exercises aim to improve cardiovascular health, muscular strength, and performance in daily living. Dynamic and static stretching exercises are used to improve mobility and reduce risk of injury. Each participant is provided with an individualized exercise plan to do at home. Exercise plan takes into account barriers to exercise and exercise capacity. Throughout the program, participants gradually increase duration of exercise from 20 minutes per day for four days per week to 60 minutes per day for five or six days a week. After the conclusion of the program, participants are encouraged to maintain an exercise duration of 60 minutes per day for five or six days a week in order to maintain weight loss.

### Cognitive-behavioral intervention

Behavioral goal setting, cognitive restructuring, self-monitoring of eating and exercise, relapse prevention, assertive communication skills, and stress management are the foci of these intervention sessions (14–16). These sessions are held throughout the Why WAIT program and conducted by a behavioral therapist or clinical psychologist. Previous clinical trials supported this modality of intervention for weight management (17, 18).

### In-person model

Traditionally, the program is delivered over 12 consecutive 2-hour weekly sessions that took place at the Joslin Diabetes Center in Boston, MA Additionally, participants are provided with handouts that include education materials, food logs, and exercise instructions and activity logs.

### Virtual model

In the virtual model, participants interact with the intervention team remotely and online. Several mobile applications (*GoToMeeting* [LogMeIn Inc., Boston, MA], *Why WAIT* [Healthimation Inc^®^, Boston, MA], and *Good Measure* [Good Measure Inc^®^, Boston, MA]) are used to track dietary and exercise interventions. Medication adjustments and group education sessions are conducted virtually and followed by text communication throughout the week (10). Each virtual visit lasts for 2 hours similar to the in-person program. Virtual demonstration of exercises is done by a RCEP. Additionally, exercise plans and instructions are available through mobile applications.

Participants are instructed on how to upload their CGM data, which is reviewed weekly by the program’s nurse practitioner and adjustments of medications are made accordingly. Cognitive-behavioral therapy took place by means of telemedicine, and additional behavioral support tools are provided through mobile applications. A detailed description of the virtual program and mobile applications was previously published (10).

### Hybrid model

The hybrid model aimed to combine the most effective parts of the virtual and in-person models, by offering two in-person sessions and 10 virtual sessions. Participants attend the first and last sessions of the 12-week program in-person, where they participate in group-education sessions, guided exercise interventions, and have their weight measured on a standardized clinic scale. In the 10-week virtual portion of the program, they use telemedicine and mobile applications, as described above.

### Study participants and design

This retrospective study included 56 participants who were enrolled in the Why WAIT program between February 2019 to April 2022. Twenty-two participants enrolled in the in-person model (iPM) of the program before COVID-19 between February 2019 and December 2019. Sixteen participants enrolled in the virtual model (VM) between April 2020 and December 2020, and eighteen participants enrolled in the hybrid model (HM) between September 2021 and April 2022. Primary endpoints for this study are changes in A1C and body weight after 12 weeks of intervention. Secondary endpoints include changes in BP, lipid parameters, number of antihyperglycemic medications, use of insulin, and percentage of glucose TIR obtained by CGM.

### Statistical analysis

Descriptive statistics were used to evaluate baseline and demographic data. All continuous variables are expressed as mean ± standard deviation (SD) or mean [95% confidence interval (CI)]. Categorical variables are expressed as percentages. Chi-square test and paired t-test were used to compare baseline characteristics and within-group differences in endpoints at 12-weeks. Quantitative differences between groups were evaluated using the Kruskal-Wallis H test. A p value of <0.05 was considered statistically significant. All analyses were performed using STATA Special Edition 15.0 for Windows^®^ (StataCorp^®^, College Station, Texas, USA 2017).

## Results

We evaluated 56 participants (age 55.9 ± 12.7 yrs; 53.6% females, 32.1% with type 1 diabetes) who enrolled in HM (n=18), VM (n=16), and iPM (n=22) of the ILI program. At baseline, there were no significant differences between groups for weight or A1C, however HM group had a lower baseline A1C (7.08 ± 0.97%) compared to VM (7.7 ± 1.3%) and iPM (7.9 ± 1.1%) but this difference between groups was not statistically significant (p=0.08). Participants in iPM had significantly higher baseline high-sensitivity C-reactive protein (hs-CRP) of 7.2 ± 6.2 mg/L compared to VM (3.0 ± 3.8 mg/L) and HM (6.4 ± 3.2 mg/L) (p=0.03 between groups). Furthermore, there were no significant differences between groups in all other parameters at baseline (duration of diabetes, BP, lipid parameters, urinary microalbumin/creatine ratio, number of antihyperglycemic medications, number of antihypertensive medications, insulin use, and glucose TIR) (**Table 1**).

**Table 1:** Baseline characteristics of participants in real-world intensive lifestyle intervention.

	All participants	In-person model (iPM)	Virtual model (VM)	Hybrid model (HM)	p Value*
**Age (years)**	56.0 (12.7)	56.3 (10.9)	58.2 (8.9)	53.4 (17.0)	0.90
**Female sex (%)**	53.6	68.2	31.2	55.6	0.08
**Type 2 diabetes (%)**	67.9	63.6	81.2	61.1	0.39
**Duration of Diabetes (years)**	16.9 (12.8)	20.2 (15.2)	14.9 (7.4)	14.6 (13.2)	0.39
**Weight (kg)**	225.9 (43.8)	104.8 (17.7)	99.2 (20.8)	102.6 (22.1)	0.23
**Body mass index (kg/m2)**	35.0 (6.4)	36.1 (5.1)	33.4 (6.3)	35.1 (7.8)	0.20
**A1C (%)**	7.6 (1.2)	7.9 (1.1)	7.7 (1.3)	7.1 (1.0)	0.08
**Systolic blood pressure (mm Hg)**	127.1 (16.9)	128.4 (15.8)	129.7 (12.5)	123.6 (20.8)	0.62
**Diastolic blood pressure (mm Hg)**	76.0 (9.8)	76.3 (10.8)	76.0 (9.8)	75.4 (8.8)	0.88
**Total cholesterol (mg/dL)**	155.5 (33.1)	158.7 (29.0)	144 (32.2)	163.5 (40.1)	0.12
**LDL-cholesterol (mg/dL)**	86.7 (29.4)	83.4 (28.7)	81.7 (27.3)	97.4 (32.5)	0.43
**HDL-cholesterol (mg/dL)**	48.5 (14.7)	51.9 (15.3)	46.0 (14.8)	45.1 (13.1)	0.48
**Triglycerides (mg/dL)**	169.9 (157.6)	173.0 (164.5)	181.1 (189.0)	149.5 (101.8)	0.80
**UACR (µg/mg)**	83.8 (214.5)	107.8 (227.5)	134.4 (314.0)	15.9 (23.0)	0.11
**hs-CRP (mg/L)**	5.3 (4.8)	7.2 (6.2)	3 (3.8)	6.4 (3.2)	0.03
**Number of diabetes medications**	2.5 (1.1)	2.5 (0.9)	2.8 (0.98)	2.1 (1.4)	0.29
**Number of antihypertensive medications**	1.1 (1.0)	1.2 (0.9)	1.06 (0.9)	0.8 (1.2)	0.27
**Time in range (%)**	74.5 (22.9)	69.0 (27.6)	77.0 (20.2)	78.0 (20.1)	0.76
**<70 mg/dl (%)**	3.1 (6.9)	4.6 (10.4)	2.5 (4.9)	1.9 (1.4)	0.54
**>180 mg/dl (%)**	22.4 (21.5)	27.9 (22.1)	17.9 (22.1)	19.9 (20.1)	0.31

Data are given as mean (SD) or %. hs-CRP, high sensitivity C-reactive protein; HDL, high-density lipoprotein; LDL, low-density lipoprotein; TDD, total daily dose of insulin; UACR, urine albumin-creatinine ratio. *Kruskal-Wallis H test or chi-square test.

Normality values: A1C <5.7%, Total cholesterol <200 mg/dL, Triglycerides <150 mg/dL, HDL-Cholesterol >50 mg/dL in women and >40 mg/dL in men, LDL-Cholesterol <100 mg/dL, UACR <30 µg/mg, hs-CRP <2 mg/L.

**Figure 1:**
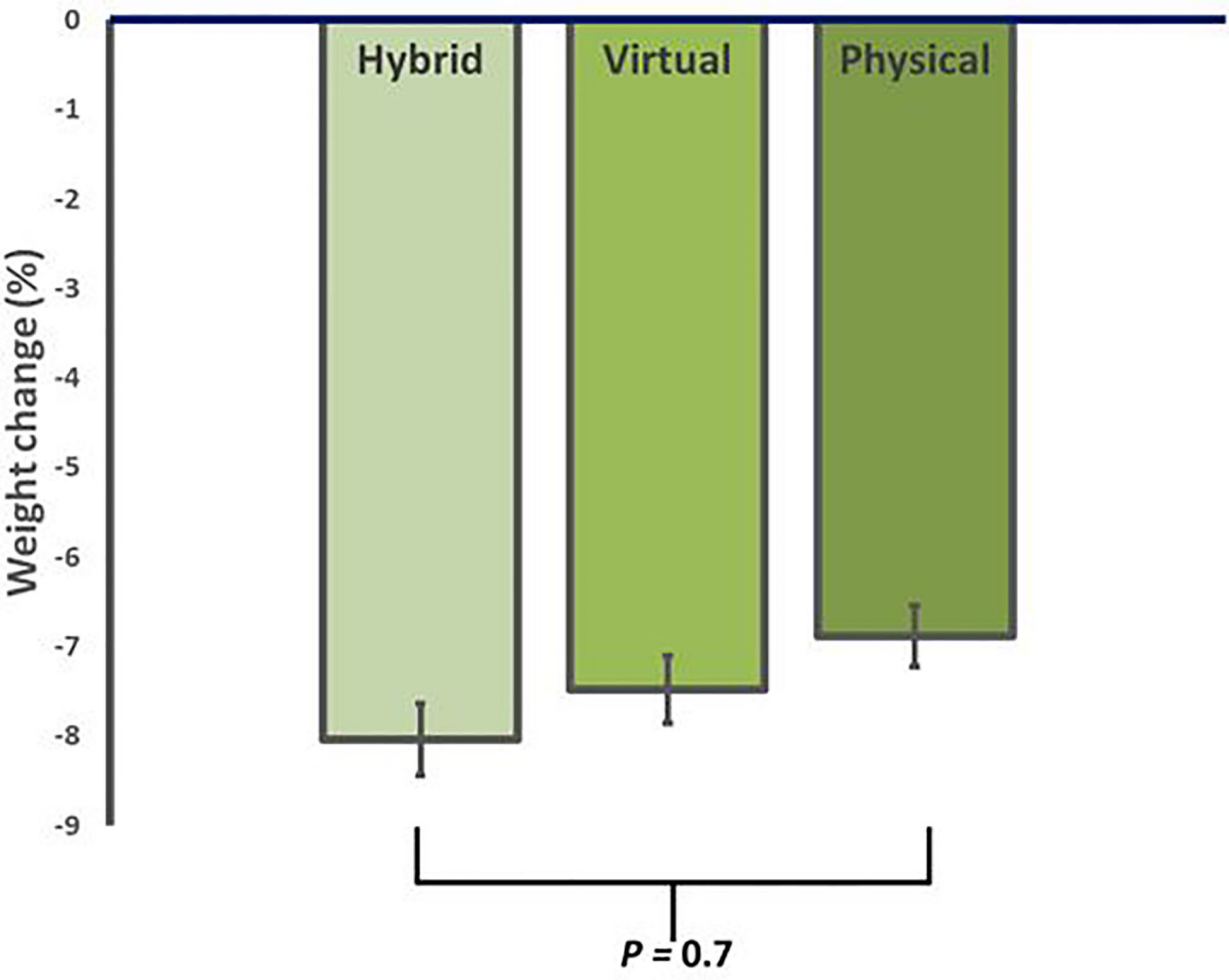
Average reduction in body weight for in-person (iPM), virtual (VM), and hybrid models (HM) after 12 weeks in a real-world intensive lifestyle intervention. All participants N = 56, iPM: n = 22, VM: n = 16, HM: n=18. p = change from baseline between iPM, VM, and HM.

After 12 weeks of ILI, participants in HM had an average weight loss of -8.2 ± 5.0 kg (95% CI, -10.8 to -5.7 kg), corresponding to -8.0 ± 4.8% (p<0.001 from baseline). Similarly, VM showed an average weight loss of -7.5 ± 3.6 kg, corresponding to -7.4 ± 3.0% (p<0.001 from baseline), while iPM showed an average weight loss of -6.9 ± 3.5 kg, corresponding to -6.6 ± 3.4% (p<0.001 from baseline) (**Table 2**). There were no significant differences in weight loss between groups (p=0.7) (**Figure 1**).

**Table 2:** Changes in metabolic and cardiovascular risk factors after 12 weeks of intensive lifestyle intervention in real-world clinical practice.

	In-person model (iPM)			Virtual model (VM)			Hybrid model (HM)			
	Baseline	12 weeks	Change from baseline	Baseline	12 weeks	Change from baseline	Baseline	12 weeks	Change from baseline	p value
**Weight (kg)**	104.8 (17.7)	97.9 (16.6)**	-6.9 (-8.5 to -5.3)	99.2 (20.8)	91.7 (18.9)**	-7.5 (-9.4 to -5.5)	102.6 (22.8)	94.4 (20.3)**	-8.2 (-10.8 to -5.7)	0.7
**Body mass index (kg/m^2^)**	36.1 (5.06)	33.8 (5.02)	-2.3 (-2.8 to -1.8)	33.4 (6.4)	31 (6)	-2.5 (-3.1 to -1.9)	35.1 (8)	32.4 (7.4)	-2.7 (-3.6 to -1.9)	0.8
**A1C (%)**	8.0 (1.1)	7.0 (0.6)*	-1.0 (-1.5 to -0.4)	7.7 (1.3)	6.7 (0.5)*	-1.0 (-1.6 to -0.4)	7.1 (1)	6.5 (1)*	-0.6 (-0.93 to -0.27)	0.6
**Systolic blood pressure (mm Hg)**	128.4 (15.8)	123.2 (11.8)	-5.1 (-11.3 to 1)	129.7 (12.5)	121.2 (10.3)	-8.4 (-19.5 to 2.6)	123.6 (20.8)	117.6 (17.6)	-6.1 (-16.5 to 4.3)	0.9
**Diastolic blood pressure (mm Hg)**	76.3 (10.8)	74.9 (8.6)	-1.4 (-6 to 3.3)	76.0 (9.8)	71.8 (7.7)	-4.2 (-9.6 to 1.3)	75.4 (8.8)	73.1 (8.4)	-2.3 (-7.8 to 3.1)	0.8
**Total cholesterol (mg/dL)**	158.8 (29)	151.5 (33)	-7.2 (-19.3 to 5)	144.2 (32.3)	127.7 (25.7)*	-16.5 (-30.4 to -2.6)	163.5 (40)	148.9 (34.5)	-14.7 (-44 to 14.8)	0.6
**LDL-cholesterol (mg/dL)**	83.4 (28.8)	81.1 (31.7)	-2.3 (-12.9 to 8.3)	81.7 (27.3)	67.9 (17.9)	-13.8 (-32.1 to 4.4)	97.4 (32.5)	81.8 (28.3)	-15.5 (-40.1 to 9)	0.6
**HDL-cholesterol (mg/dL)**	51.9 (15.3)	51.9 (16.6)	0 (-3.2 to 3.2)	46.0 (14.9)	48.0 (14.7)	2.0 (-1 to 5)	45.1 (13.1)	52.3 (16.3)*	7.1 (1.1 to 13.2)	0.8
**Triglycerides (mg/dL)**	173.0 (164.5)	119.5 (76.3)	-53.6 (-117.4 to 10.3)	181.1 (189.4)	121.1 (81.5)	-60.0 (-125.5 to 5.5)	149.5 (101.8)	101.8 (65.9)*	-47.7 (-91.9 to -3.5)	0.7
**Urinary microalbumine/creatinine ratio (µg/mg)**	107.8 (227.5)	36.1 (46)	-71.6 (-177.7 to 34.4)	134.5 (314.7)	57.2 (131.4)	-77.3 (-218.4 to 63.9)	15.9 (23.3)	6.8 (9.1)	-9 (-22.7 to 4.7)	1.0
**CRP-HS (mg/L)**	7.3 (6.2)	5.3 (3.3)	-2.0 (-2.0 to 5.9)	1.8 (1.8)	1.9 (2.2)	0.1 (-1.1 to 1.3)	6.4 (3.2)	4.3 (3.2)	-2.0 (-4.1 to.1)	0.5
**Number of diabetes medications**	2.5 (1)	1.8 (1)**	-0.8 (-1.1 to.4)	2.8 (1)	1.9 (1)**	-0.9 (-1.3 to -0.6)	2.1 (1.4)	1.7 (1.3)	-0.4 (-0.9 to 0.0)	0.1
**Number of antihypertensive medications**	1.3 (0.99)	1.0 (1)*	-0.2 (-0.3 to -0.007)	1.0 (0.2)	1.0 (0.2)	-0.06 (-0.2 to 0.1)	0.8 (1.1)	0.7 (1)	-0.1 (-0.3 to 0.1)	0.8
**T2D patients on insulin (%)**	57.1	21.4*	-35.7	46.2	7.7*	-38.5	18.2	0.0	-18.2	0.5
**Time in range (%)**	69.0 (27.6)	72.3 (22.7)	5 (-5 to 11.8)	77.0 (20.2)	87.3 (14.0)	10.3 (-10.9 to 21.7)	78.0 (20.1)	82.1 (14.8)	4.1 (-14.8 to 9.6)	0.4
**< 70 mg/dl (%)**	4.6 (10.4)	1.0 (1.6)	-1.0 (-3.0 to 0.5)	2.5 (4.9)	0.6 (1.8)	-1.5 (-3.8 to 0.7)	1.9 (1.4)	2.8 (5.0)	0.9 (-1.8 to 3.7)	0.8
**> 180 mg/dl (%)**	27.9 (22.1)	46 (90)	31 (-15 to 7)	17.9 (22.1)	12 (13)	-3 (-16 to 8)	19.9 (20.1)	14.0 (15.4)	8 (-10 to -1)	0.2

Data are given as mean (SD) or mean (95% CI). P value = change from baseline between iPM, VM and HM. HDL, high-density lipoprotein; LDL, low-density lipoprotein. **p < 0.001 compared with baseline. *p < 0.05 compared with baseline.

Normality values: A1C <5.7%, Total cholesterol <200 mg/dL, Triglycerides <150 mg/dL, HDL-Cholesterol >50 mg/dL in women and >40 mg/dL in men, LDL-Cholesterol <100 mg/dL, UACR <30 µg/mg, hs-CRP <2 mg/L.

**Figure 2:**
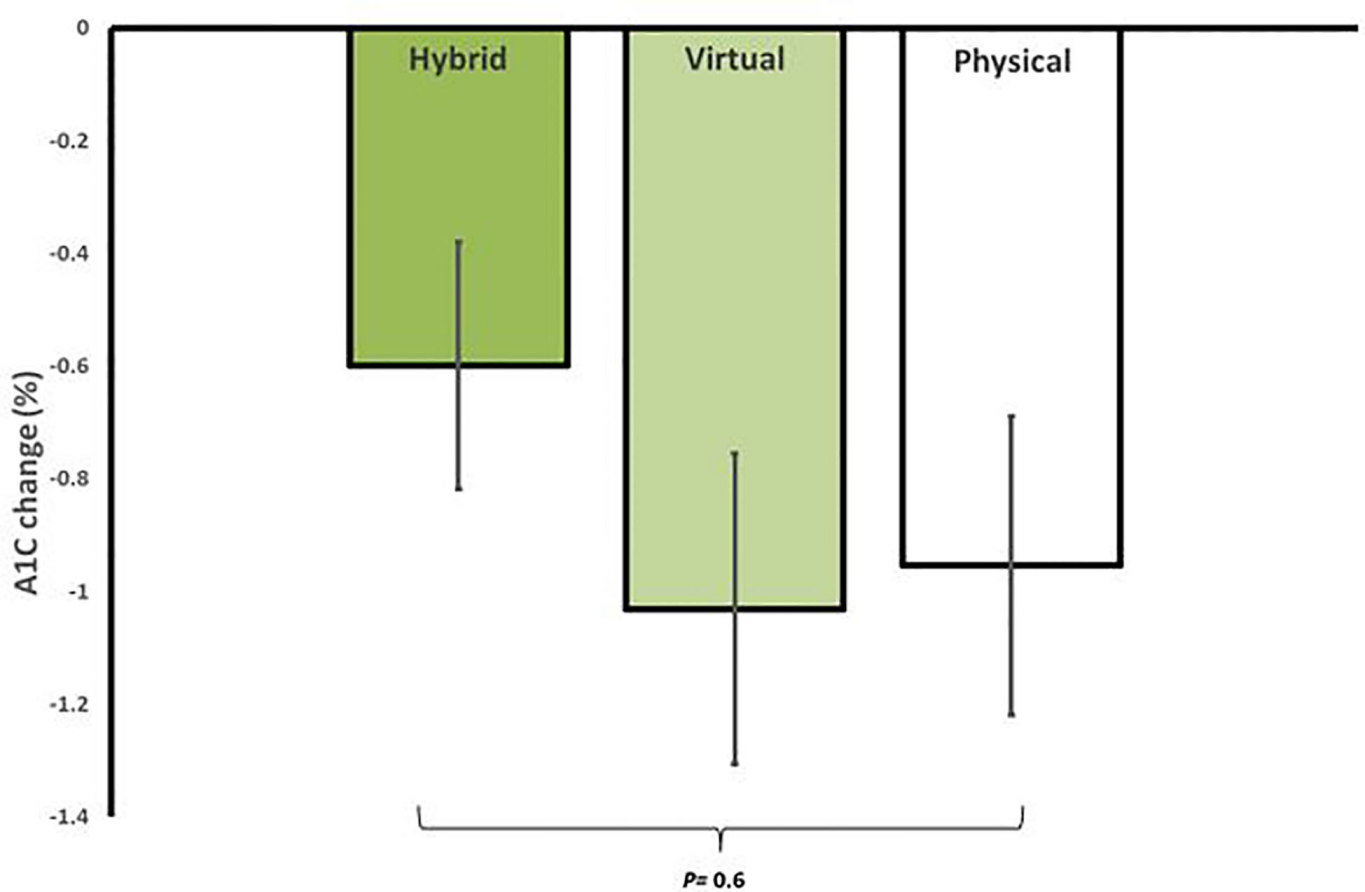
Average reduction in A1C for in-person (iPM), virtual (VM), and hybrid models (HM) after 12 weeks in a real-world intensive lifestyle intervention. All participants N = 56, iPM: n = 22, VM: n = 16, HM: n=18. p = change from baseline between iPM, VM, and HM.

In regard to glycemic control, all groups showed significant improvements from baseline. HM had an average reduction in A1C of -0.6 ± 0.6% (95% CI, -0.9 to -0.3, p=0.002), VM had an average reduction in A1C of -1.0 ± 1.1% (95% CI, -1.6 to -0.4, p=0.002), and iPM had an average reduction in A1C of -1.0 ± 1.2% (95% CI, -1.5 to -0.5, p=0.001). There were no significant differences in A1C reductions between groups (p=0.6) (**Figure 2**). Furthermore, among participants with T2D treated with insulin, 100% (n=2) in HM, 83.3% (n=5) in VM, and 62.5% (n=5) in iPM stopped insulin at 12 weeks. Reductions in the number of antihyperglycemic medications were not significant between groups, with participants from HM reducing their meds by 17.6%, VM by 36.1%, and iPM by 28.8% (p=0.2).

In evaluating CGM data at week 12, participants in HM had an average glucose TIR of 83 ± 15%, compared to 87 ± 14% in VM and 72 ± 23% in iPM. There was no statistical significance between groups (p=0.2). There were no significant differences between groups for percentage of time in low glucose, defined as < 70 mg/dl (p=0.5), or percentage of time in high glucose, defined as > 180 mg/dl (p=0.2). Lastly, all groups showed improvements in BP, lipid profile, and number of antihypertensive medications at 12 weeks, with no statistically significant differences between groups (**Table 2**).

## Discussion

Intensive lifestyle modification has been cornerstone in the management of diabetes and obesity in the last few decades (19). The Why WAIT program is a 12-week multidisciplinary intensive lifestyle intervention program that showed long-term maintenance of weight loss for up to 10 years (8, 9). The in-person model of the program has been offered to patients at Joslin Diabetes Center in Boston, MA since 2005. In 2020, the COVID-19 pandemic limited access to face-to-face interaction with the multidisciplinary team and necessitated the need for a virtual model. After 2 years of a totally virtual model of intervention, the program was offered as a hybrid model; giving patients the enhanced flexibility of both in-person and virtual modalities.

In a previous study, we noticed equal efficacy between the virtual model of the program and the in-person model with similar benefits in weight and A1C reduction, and improvement in cardiovascular risk factors (10). However, a fully virtual model has several limitations. First, the inability to measure participants’ body composition, visceral fat, or to evaluate physical fitness and exercise ability. Second, participants had to complete their weekly exercise assignments at home, since they were unable access Joslin’s gymnasium (10). Furthermore, current literature discussing virtual ILI showed that patients are more likely to enroll in such virtual programs but less likely to attend all sessions (20). A key strength of the in-person model is accountability through weekly face-to-face encounters with the Why WAIT team. Additionally, the in-person program allows for standardized weight measurements taken by the team on the same scale. The hybrid model was designed to overcome a few of the challenges associated with the virtual program while keeping most of its demonstrated strength. In the in-person-component of the hybrid model, participants are properly evaluated and meet in the first session and last session with the intervention team. The virtual component of the hybrid model allows participants to perform most aspects of the program on their own schedule and from the convenience of their own home without the need for scheduling their visits or enduring timely and costly commutes to the clinic. They can also record their diet and exercise logs electronically through mobile applications, which make it easier and more reliable. Furthermore, participants have access to all the exercise and behavioral education tools on the virtual platform.

This new model resulted in statistically equivalent improvements in body weight, A1C and cardiovascular risk factors. Participants in HM were able to achieve an average weight loss 8.0% of their baseline body weight. Significant reductions in A1C levels were observed in all three groups, with participants in HM achieving A1C reduction of -0.6 ± 0.6% after 12 weeks of intervention. Changes in weight loss and A1C were not significantly different between groups. Furthermore, since long-term data of the in-person program showed improvements in cardiovascular risk factors (9), we hope to assess long-term data of the virtual and hybrid programs once available.

Additionally, CGM use helped Why WAIT team to track trends in glycemic patterns and adjust medications, exercise plan, and nutrition plan appropriately. By the end of the program, CGM download showed no statistically significant differences between groups, but it is worth mentioning that participants in HM were able to maintain greater percent TIR at 12 weeks compared to iPM. These findings are consistent with our previous observation comparing VM to iPM. This may be attributed to ability of the Why WAIT team to accurately track glucose readings and frequently adjust medications (10). Improvement in cardiovascular risk factors including blood pressure, lipid profile, urinary microalbumin and CRP were not different between groups. Reduction in the number diabetes medications and their doses were also not different between the 3 groups.

While technology implementation in medicine is helpful, it always carries its challenges especially when it comes to patients’ perspective. The main challenge is digital education and use of newer technology like wireless, cellular, or Bluetooth devices. The hybrid model offers the advantage of in-person training during the first visit, ensuring participants’ ability to conduct the rest of the program efficiently from home. However, age and socioeconomic factors such as education and income, which might affect adherence to technology-based aspects of the programs, were not assessed. Additionally, the hybrid program’s scalability makes it feasible to offer the program to a wider geographic area and include participants who have limited ability to commute for long distances on a weekly basis. Although costs (direct or indirect) associated with each program were not assessed in this study, a hybrid model is potentially less costly after eliminating the commuting and parking costs. No show and cancellations were also minimized. Further research is required to evaluate the health economics and cost-effectiveness of the 3 different models.

## Conclusion

Looking toward post-COVID era, where patients are prioritizing flexibility in their daily lives as well as making use of more digital health options in their health care, the hybrid model provides patients with the option of participating in the program in a way that adapts to the intricacies of a post-pandemic era. While providing more flexibility than the in-person model and greater accountability than the virtual model, the hybrid model does not jeopardize the face-to-face interaction with the multidisciplinary team, which remains an essential component of ILI programs. While the virtual and in-person models have their unique benefits and drawbacks, the combination of them, which culminated in the hybrid model, created a synergetic model that enhances delivery of a multidisciplinary program. It also prioritizes patients’ need for flexibility, keeps patients accountable, and allows for accurate recording of measures of interest, while giving participants an equally effective intervention that reduces their body weight and improves their A1C by the same highly specialized multidisciplinary team.

## Data availability statement

The raw data supporting the conclusions of this article will be made available by the authors, without undue reservation.

## Ethics statement

The studies involving human participants were reviewed and approved by Institutional Review Board of Joslin Diabetes Center, STUDY00000133. Written informed consent for participation was not required for this study in accordance with the national legislation and the institutional requirements.

## Author contributions

Conceptualization, SD, MA-B and OH. Data curation, SD, MA-B, TS, CK, JS, AK, KK, RC and OH. Formal analysis, SD, MA-B, TS, AK, KK, RC and OH. Funding acquisition, OH. Investigation, SD, MA-B, TS, CK, JS, CJ, JV, CM, JB, AK, KK, RC, CD and OH. Methodology, SD, MA-B, TS and OH. Project administration, SD and OH. Resources, SD, MA-B and OH. Supervision, SD and OH. Validation, SD, MA-B and OH. Visualization, SD, MA-B, AK, KK, RC and OH. Writing – original draft, SD, MA-B, TS and OH. Writing – review & editing, SD, MA-B, TS, CK, JS, CJ, JV, CM, JB, AK, KK, RC, CD and OH. All authors contributed to the article and approved the submitted version.
